# Prophylaxis in the Field of the Dental Surgeon

**Published:** 1891-06

**Authors:** Chas. B. Atkinson

**Affiliations:** New York


### PROPHYLAXIS IN THE FIELD OF THE DENTAL SURGEON.[1]

BY CHAS. B. ATKINSON, D.D.S., NEW YORK.

Prophylaxis presents four closely-related and two attendant aspects for consideration.

1. Prevention, properly a broad effort of education to teach to avoid.

2. Diet, a means of preparation of the system to assist prevention.

3. Hygiene, a regulation of circumstances closely governing.

4. Regimen, ruling of use of system, food, article, and circumstance, under the instruction of the preceding aspects; add to these operative and medicinal interference in the progress of disordered and diseased conditions, and the breadth of prophylaxis is before us.

Of prophylaxis, it is first required to subdue the ignorance of the public at large as to hygiene, diet, and personal care, so that teeth may be supported and aided to attain, and be maintained in, a healthful condition.

Publication under society endorsement, considering the feeding of mothers and children; exercise of the teeth, means of securing their cleanliness, and the commonly-occurring conditions resulting from accident, disease, and carelessness; their possibilities of repair, and the lines of care which control prevention of deficient function of the teeth, all come within the scope of this effort of prophylaxis.

A diet for mothers during gestation—indeed, suitable for any adult human organization—would include lean meat, fibre for muscle-

---

[1] Read before the New Jersey State Dental Society at the annual meeting, July, 1890.

building; starch producers for sugar, for carbon and acid reaction; fats in limited quantities, for carbon and lubrication; cereals (graham, rye, corn-bread, oatmeal), for bone formation; coffee and tea in limited quantities and of moderate strength, taken without milk, as digestive and nerve stimulants; turnips, spinach, cabbage, for intestinal residue; salt, of necessity; pure water, as a diluent; ripe fruits.

For babies artificially fed, some one or more acceptable manufactured food, barley-water, browned wheat-flour, with the addition of beef-juice expressed raw.

For children when weaned, barley and milk, lean meat minced fine, and particularly mutton, toasted bread, and crackers.

This is advanced with the hope that it may draw more light on the question of specific diet.

Hygiene and regimen call attention to general health of the body, indications in which connection are fresh air and exercise, regularly taken; frequent cool bathing, using little soap, but plenty of rubbing. The feet, as a rule, have but indifferent exercise, confined as they are by the coverings fashion dictates, and therefore they should receive especial attention in rubbing. It has been urged that rubbing with the hand from the extremities towards the trunk aids the return of the blood laden with effete oxygen and carbonic acid gas.

Only sufficient clothing for comfort should be worn (perhaps wool next the skin), particularly about the chest, armpits, abdomen, and knees; not too much about the throat. Further than this, thorough chewing is called for; careful brushing of the teeth, with powder occasionally, but regularly with some antisepticized soap, particularly at night (Buchan's carbolic tooth-soap is recommended); efficient daily use of floss silk; rinsing, gargling, spraying, or syringing with antiseptic preparations, such as salt-and-water, hydronaphthol solution, bichloride of mercury solution, peroxide of hydrogen.

These general phases prepare the way for attention to the systemic indications properly embraced in the professional employment of the dental surgeon, whether it be reflex action or inception of dental disease.

Disasters being subject to influence external to the body, are removed from consideration under the title of this paper. Nevertheless a certain degree of loss of tissue requires substitution to fulfil adequate prophylaxis. Inefficiency follows on any infraction of nature's design; but after this reaches a certain degree, substitution is imperative to secure against progressive destruction beyond the power of unaided systemic repair or endurance.

Caries of teeth call loudly for prophylactic attention ; dyspepsia, catarrh (general and local), irregularity, constitutional taints and defects (influencing structure of tooth-substance), present important fields for investigation and the administration of efficient preventives, the control and subjection of the inducing element being the only positive, lasting prophylactic.

Mere temporizing with local manifestations of systemic derangement, although prophylactic, is so but in degree, and merely interrupts the expression of disordered function.

Prophylaxis should be more than this. The filling of teeth, their extraction and treatment, are all prophylactic as defending from more serious consequences, but not necessarily complete as an effort in that direction.

The filling of carious teeth in the mouth of a dyspeptic, for instance, is but a secondary phase of the prophylaxis indicated, and the lapse of time will develop further need for such effort again and again, until the eradication of the recognized predisposing antecedent, which, disregarding fractures, is believed is always one of either remote or immediate systemic aberration.

The filling naturally is indicated as an essential factor (as far as present knowledge guides us), but to this is added such further measures as are needed to complete the partial purpose of the filling.

Attention is directed to a treatment of oxyphosphate cement some time since presented in detail by the writer, which is believed to offer inherent medicinal advantages aside from an increased resistance to solution, which is believed is secured by the system.[1]

Many times all further that may be presented to mind is instruction in the local care of the teeth and mouth.

In this connection, attention is naturally directed to the sanitary conditions influencing catarrh (as expressing the wide range of mucous tissue, debility, and consequent congestion attendant on various unsanitary conditions).

Also to adenoid growths, inducing mouth-breathing, with the attendant abnormal lung, nerve, and muscle action and inaction, and also of the common result in this condition of irregularity, which induces mal-occlusion, removing the possibility of proper exercise

---

[1] *Ohio Journal*, January, 1890.

of the teeth in chewing, which all come within the scope of dental prophylaxis.

In connection with sanitary conditions may be noted a perhaps not prevalent source of the propagation of disease, but nevertheless a matter of moment in itself, although one that is probably efficiently managed by all dentists, properly so called, and this is the absolute cleanliness of the instruments and appliances and hands brought into contact with the patient.

This matter has been lengthily considered recently by two medical gentlemen,[1] and the simple but effective method proposed of a receptable containing water kept boiling, being within easy reach of the operating-chair, into which all instruments when used may be dropped and effectually sterilized.

It has the advantage of not discoloring steel instruments, as bichloride solution does, and when wiped dry they are *clean* and need only an occasional buffing, instead of the continual polishing the use of a bichloride solution necessitates.

A very urgent call for prophylaxis is heard from the sick-room. Prolonged invalidism is generally productive of extensive caries of teeth, and not unattended by several other grave lesions of the oral cavity.

A reciprocative irritation between the mouth and stomach and the whole mucous tract induces mutual pernicious influence one upon the other and back again to an aggravated condition which, were either, or both, controlled, would much lessen the gravity of many prolonged diseases, not to say avoid new complications, as, for instance, a carious tooth proceeding to death of the pulp, and abscess followed by necrosis of the maxilla, a frequent result of neglected abscessed pulps, perhaps the most frequent antecedent of necrosed maxillæ, although the neglect of a fractured alveolus (fractured in careless extraction) is also a prolific source of this condition.

What may we do to ward off disaster to the teeth and mouth during illness? The patient, his attendant or nurse, should be taught to pass the floss, if at all feasible, carefully, gently, yet thoroughly, between every space, once at least daily. This should form part of a nurse's training when preparing themselves for their vocation. The floss should be rendered aseptic, as also the fingers of the nurse, as carefully as when attending to a surgical dressing. The use of a brush is so awkward in the hands of another person

---

[1] Dr. Wight, before the Brooklyn Dental Society, in January last; Dr. Bulkley before the Odontological Society of New York, in April last, 1890.

that the patient would be likely to resent it. The floss can be kindly and acceptably used by another person, and should be. Following the floss, antiseptic mouth-washes or sprays would be very effective in securing the patient from serious disaster, even in prolonged sickness. It is desired to protest against the use of orris-root or other fermentable substances in tooth-powder, although orris-root has been distinguished as a component of tooth-powder for long years, and it is to-day prescribed by physicians and in sickness. It is time that it be replaced by some efficient non-fermentable substance.

Systemic disorders, as dyspepsia, general catarrh, kidney and lung diseases, rheumatism,—all in themselves results of malassimilation,—furnish opportunity for the incitement of pyorrhœa alveolaris,—a disease primarily induced (in its restricted local aspect) by a lowered tone of gum-tissue and blood-vessel coats, permitting congestion to present an initial stage, followed by distention, disruption, and solution, until suppuration removes the myxomatous tissue in such quantity as to call upon the system for a sufficiently accelerated blood-supply to terminate the local manifestation, usually after the loss of all the teeth and alveolar processes (which have been the framework about which the loose, coarse gum-tissue was formed).

Peripheral relaxation is a reasonable sequence of deficient central nerve-tension. Malassimilation being at the seat of the beginning of nutrition, presents a constant factor in disease in every one of its aspects.

Were proper food properly assimilated, disease would be unknown.

The study of individual requirement based upon individual structure, endowment, and environment, embracing physical, mental, and circumstantial characteristics, seems to be the line of investigation that will present facts upon which to predicate acts of guidance (advice), which offers the highest expression of professional service, prophylaxis.

This advances dentistry to an ideal position.

In the mean time we must do the best we can, and make efforts looking to this ideal result in constant investigation and the continual advancement of our educational plan, and by no means the least effort we may make to this end will be the persistent education of the general public to an understanding of conservative health, hygienic measures, and an intelligent comprehension of the possibilities of repair within the ability of modern dentistry, looking to an eradication of disease, gradually but surely.

